# Efficacy of Manual Therapy in Temporomandibular Joint Disorders and Its Medium-and Long-Term Effects on Pain and Maximum Mouth Opening: A Systematic Review and Meta-Analysis

**DOI:** 10.3390/jcm9113404

**Published:** 2020-10-23

**Authors:** Andres Herrera-Valencia, Maria Ruiz-Muñoz, Jaime Martin-Martin, Antonio Cuesta-Vargas, Manuel González-Sánchez

**Affiliations:** 1 Department of Physiotherapy, Faculty of Health Sciences, University of Málaga, 29071 Málaga, Spain; andresherrval@gmail.com (A.H.-V.); acuesta@uma.es (A.C.-V.); mgsa23@uma.es (M.G.-S.); 2 Department of Nursing and Podiatry, Faculty of Health Sciences, University of Málaga, 29071 Málaga, Spain; 3 Institute of Biomedicine of Málaga (IBIMA), 29010 Málaga, Spain; jaimemartinmartin@uma.es; 4 Department of Human Anatomy, Legal Medicine and History of Science, Faculty of Medicine, University of Málaga, 29010 Málaga, Spain; 5 School of Clinical Sciences of the Faculty of Health, Queensland University of Technology, Brisbane QLD 4000, Australia

**Keywords:** manual therapy, temporomandibular, joint, pain, review

## Abstract

The aim of this study was to conduct a systematic review of the medium- and long-term efficacy of manual therapy for temporomandibular joint disorders, alone or in combination with therapeutic exercise. Information was compiled from the PubMed, SCOPUS, Cochrane, SciELO and PEDro databases. The inclusion criteria were established: randomized controlled trials only; participants must present any kind of temporomandibular disorder; the treatments must include manual therapy in at least one of the experimental groups; a minimum of 3 months of follow-up; pain must be one of the primary or secondary outcomes; and the article must be available in English, Spanish, Italian, Portuguese or French. Six documents that fulfilled all the criteria were obtained for analysis, two of them considered low quality and four considered high quality. A significant improvement in pain and mouth opening compared to baseline was observed after manual therapy treatment. Manual therapy seems to be an effective treatment for temporomandibular disorders in the medium term, although the effect appears to decrease over time. However, when complemented with therapeutic exercise, these effects can be maintained in the long term. This review underlines the importance of manual therapy and therapeutic exercise for the medium- and long-term treatment of temporomandibular joint disorders in daily practice.

## 1. Introduction

The temporomandibular joint (TMJ) consists of two synovial joints that work together. However, this unique trait presents certain problems, and the treatment of these issues is often complex. Disorders related to the masticatory system are designated “temporomandibular disorders” (TMD), which are considered the main musculoskeletal cause of orofacial pain [1,2]. A large proportion of the global population is affected by TMD, with an estimated 25% of adults presenting signs and/or symptoms [3]. TMD affects females at a rate between 1.5 and 2.5 times higher than males [4]. The classification of TMD can be complex, as these disorders can be caused by articular problems, a displaced disc, hypermobility syndrome or masticatory muscle problems, among others [5]. Disc displacement is considered to be a common cause of pain [6] and can be classified into two groups: disc displacement with reduction, when the disc returns to its original state once the movement is completed, and disk displacement without reduction, when the disc is unable to return to its normal position [5]. Disc displacement with reduction does not typically include pain or mouth opening limitation, which usually leads to a lack of early treatment [7], whereas disc displacement without reduction often follows a course with pain and mouth opening limitation [8]. Pain is often a decisive outcome in TMD, even if it does not result directly from the physiopathology itself, and it often ends up affecting the function and quality of life of patients [9].

The treatment of TMD is usually addressed with several therapies, including splints, psychotherapy [10,11], acupuncture [12], and various physiotherapy techniques such as transcutaneous electrical nerve stimulation (TENS), lasers, massage therapy [13], joint mobilization and therapeutic exercise (TE) [14].

Numerous studies have researched the effectiveness of TE in different subacute and chronic musculoskeletal pathologies [12,13,14,15], and TE is often recommended for TMD in conjunction with manual therapy (MT) [9,16]. Although the effectiveness of these therapies has been studied in the short and mediumterm, returning diverse results [16,17], no systematic reviews have analyzed the medium- and long-term effects of MT and TE for TMD.

Therefore, the main objective of this study was to conduct a systematic review and meta-analysis of the medium- and long-term efficacy of MT for TMD, alone or in combination with TE.

## 2. Methods

A systematic review of the scientific literature was carried out following the Preferred Reporting Items for Systematic Reviews and Meta-Analyses (PRISMA) guidelines. This review was registered in the International Prospective Register of Systematic Reviews (PROSPERO) under the identification number CRD42019133213.

### 2.1. Search Strategy

Information was compiled from the PubMed, SCOPUS, Cochrane, SciELO and PEDro databases using search terms from the Medical Subject Headings (MeSH) vocabulary and included documents published up to 31 July 2020. The keywords used for the search were “manual therapy”, “manipulative therapy”, “temporomandibular joint”, “temporomandibular disorders”, “temporomandibular joint disorders”, “TMJ” and “TMD”.

### 2.2. Study Selection

After obtaining the articles, duplicate articles and those that did not meet the established criteria were removed, starting by reading their titles. Afterwards, abstract readings were carried out, and subsequently, full-text readings were performed. All articles were individually assessed by two researchers, and any disagreements were resolved by consensus.

### 2.3. Inclusion and Exclusion Criteria

To gather all relevant data, the following inclusion criteria were established: (1) randomized controlled trials (RCTs) only; (2) participants must present any kind of TMD; (3) treatments must include MT in at least one of the experimental groups, complemented or not with TE; (4) a minimum of 3 months of follow-up; (5) pain must be one of the primary or secondary outcomes; and (6) the article must be available in English, Spanish, Italian, Portuguese or French. The following exclusion criteria were established: (1) neurological patients; (2) patients with a history of traumatic damage to their temporomandibular region; and (3) studies focused on bruxism or sleep apnea.

### 2.4. Outcomes

The most frequently reported primary outcome in the analyzed studies was pain, which was usually measured with the visual analogue scale (VAS). This scale represents the pain intensity perceived by the patient and consists of a 10cm line on which patients grade their pain, where 0 represents a total absence of pain and 10 is the worst imaginable pain. Results from articles that did not use the VAS to measure pain were translated into percentages in order to standardize and compare measurements. The most frequent secondary outcomes were maximum mouth opening (MMO), measured in millimeters between the central incisors; the range of movement (ROM); and disability, which was measured through different questionnaires.

In order to provide a better estimate of the effects of the intervention, a meta-analysis was performed in an attempt to identify the TMJ outcomes caused by MT and TE for TMD. The main variables of the analysis were the magnitude of the effect size for the change in pain perceived by the patient, and the MMO over time. The minimum number of analyzable results agreed for performing the meta-analysis for a variable in a measurement period was 5 results.

## 3. Results

### 3.1. Study Selection

**Figure 1:**
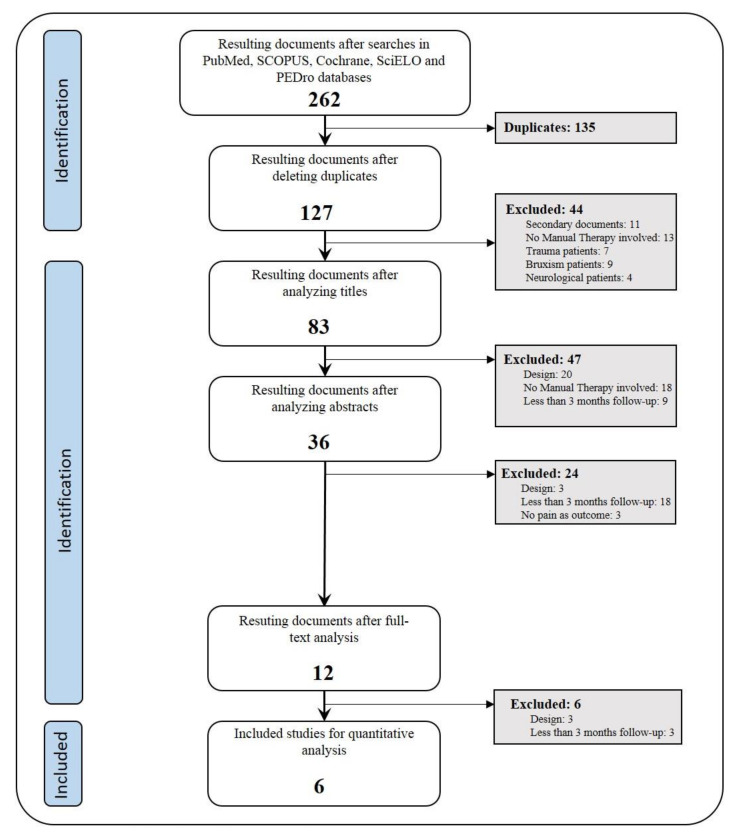
Search flow chart and documents selection.

After carrying out searches in the PubMed, SCOPUS, Cochrane, SciELO and PEDro databases using the previously stated criteria, 262 articles were obtained. Of these, 135 were excluded, as they were duplicates. The exclusion criteria were applied at every step within the process of the identification (Figure 1) of the 127 remaining documents. Filtering was carried out by two researchers, and discrepancies were resolved by consensus. Finally, six documents that fulfilled all the criteria were obtained for analysis.

### 3.2. Methodological Quality

The quality of the RCTs was measured according to the PEDro scale. This scale consists of 11 items that are answered with “yes” or “no” responses based on whether they are fulfilled or not. The score is established over 10 points, as the first item is not counted towards the final score. Documents with a score ≥6 were classified as high quality, and those with a score <6, as low quality.

Following the instructions of the PEDro scale, the articles included in this analysis scored between 4 [18] and 8 [19,20,21], with two considered low-quality studies [18,22] and four considered high quality (Table 1).

None of the studies fulfilled the blinded therapist item (Item 5), although all of them fulfilled randomized allocation (Item 1), similar baseline measurements (Item 3), between-group comparisons (Item 9), and point measures and measures of variability (Item 10). Only one of the studies [19] used blinded subjects (Item 4).

In total, the analysis included 304 people who suffered from TMD, 233 of whom were women (76.64%). The average subject age was 41.5 years. The studied pathologies included mouth opening pain [18,19,21,22], mouth opening limitation [19,20,22], myofascial symptoms [14,18,19], non-reducing disc displacement [19,20] and chronic migraine [14].

The treatments employed in the studies were diverse. The most common treatment was TE, used in every study except one [19]. Other techniques that were employed in more than one study were caudal mobilization of the TMJ [19], health education and good habits communicated during the session by the clinical therapist, and manual treatment of the temporal and masseter muscles, which were all used in three out of the six articles. The following additional treatments were used in only one of the studies: cervical region treatment and TMJ neurodynamics [14], TMJ manipulation [19], botulinum toxin injections and manual pressure on craniocervical coordination centers [18], lateral and medial pterygoid muscle and sphenopalatine ganglion treatments [21], and Michigan splints.

Regarding the frequency of sessions, two sessions per week was most common, performed in three out of the six studies. Among the remaining studies, one study included one session every two weeks [19], and the other two [14,18] did not clarify the frequency. The treatment duration showed a greater variation, ranging from 2–4 weeks [20] up to 18 weeks [19]. The total number of sessions also presented great variability, from 3 (±1) sessions [18] up to 24 [21], giving a median of 9.5 sessions (Table 2).

The most frequent outcomes were pain, assessed using the VAS, and active MMO, measured in millimeters, both of which were included in all of the articles. Two studies measured the pain pressure threshold (PPT) in the temporal and masseter muscles [14,20], and two recorded the passive MMO [20,21]. The remaining outcomes appeared in only one of the studies.

One study [19] conducted a 4-month follow-up, two studies [20,21] had a 12-month follow-up, and the rest conducted a 3-month follow-up.

In every article, a significant improvement in pain (measured with the VAS) and MMO compared to baseline was observed after MT for TMJ. Comparing the effects of MT, TE and education, the differences in pain and MMO seemed to be non-significant in the medium term (3–6 months) [19,20,21].

When comparing the effect of MT for TMJ to other therapies, some differences were observed. Compared to MT in the cervical region, a significant reduction in pain was found from 3 months onwards, as well as a significant increase in MMO until 3-month follow-up [14]. Comparing treatment with manual pressure on cranio-cervical coordination centers to botulinum toxin injection, no significant differences were found during the follow-up, except for laterotrusion movements, which improved more after MT treatment [18]. The use of Michigan splints showed similar results, whether it was complemented with MT and TE or not, except when measuring active MMO, in which case a greater improvement was found when MT and TE were also used (Table 3).

**Figure 2:**
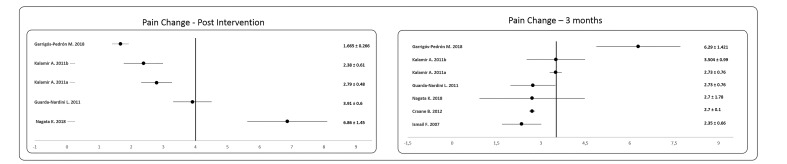
Forest Plot of the changes in pain after the intervention and three months from the baseline.

Figure 2 shows a forest plot derived from the meta-analysis performed on the change experienced in the pain variable, immediately after the intervention as well as at three months from the baseline. It is observed how immediately after the intervention, the mean improvement in the pain variable was around 4/10 points, although the range of improvement was very wide depending on the study consulted (Figure 2). However, when the results were analyzed at 3 months from the start, the average improvement remained at around 4/10, but the results were more homogeneous when comparing the different studies.

**Figure 3:**
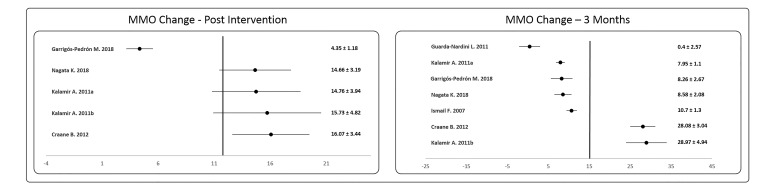
Forest plot of the changes in maximum mouth opening (MMO) after the intervention and three months form the baseline.

On the other hand, Figure 3 shows the changes experienced in the MMO both at the end of the intervention and at three months from the baseline. At the first time of measurement (after the intervention), it is observed how the average improvement of almost all the groups was close to 15 mm, with the exception of the Garrigós-Pedrón study [14], where an improvement of 4.35 mm is observed, which makes the trend line be to the left of the vast majority of the studies analyzed. When the results are analyzed at three months, it is observed that five of the seven results analyzed showed a failure to maintain the improvement experienced after the intervention, while the studies by Craane B. [20] and Kalamir A. [21] not only show maintenance of the improvement but an almost doubling of it. The reason why these differences in results are observed between the different studies at 3 months may be the types of therapeutic exercise used.

## 4. Discussion

The main objective of this review was to analyze the results of the available literature on the treatment of TMJ using MT, alone or in combination with TE, after medium- and long-term follow-up. Overall, the results suggest that MT is effective for the treatment of pain and mouth opening limitation in the medium and long term, but there are few significant differences when compared to other analyzed therapies. When combined with TE, better results are obtained, and the effects of therapy tend to last longer. On this basis, it can be concluded that the objective of the review was accomplished.

### 4.1. Pain

The results for this outcome improved when MT was used as a treatment for TMD. In the short term, pain was decreased as a result of every therapy analyzed, those being MT on the cervical region [14], manipulative techniques [19], botulinum toxin injection [18], manual pressure on cranio-cervical coordination centers [18] and MT on the TMJ. Studies that assessed other techniques, such as TE or education, did not report post-treatment results, as these techniques have no important immediate results.

In the medium term, the improvements resulting from MT on the TMJ seemed to be maintained. The other therapies included in this review also maintained their effects, except for MT in the cervical region [14]. In this study, treatments were performed on subjects with TMD of orofacial origin only, which may explain the obtained results. However, it is remarkable that the initial changes were maintained through the first 6 weeks of follow up.

When analyzing the long-term effects on pain, the effects of MT on the TMJ alone appear to diminish over time; however, the effects are maintained if MT is complemented with TE [21]. Taken together, these results emphasize the importance of TE for the long-term preservation of the desirable effects, as previously studied for other musculoskeletal pathologies with moderate evidence results [23,24]. Comparing treatments based on MT and TE to ones focused on education showed that both led to improvements in pain, with no significant differences between them [18]. These results were maintained over 13 months of follow-up, emphasizing the importance of encouraging healthy habits in order to maintain the beneficial effects.

### 4.2. Mouth Opening

The results obtained for MMO are similar to those for pain. Short-term, manipulative therapy with TE [19] caused a significant increase in MMO compared to TE only; the changes are only possible to observe after the treatment. It is important to point out that in this study, MT was performed until a certain MMO was achieved, meaning that patients would unavoidably maintain this MMO after treatment.

The medium-term results from treatment with MT and TE showed benefits. The use of the Michigan splint [22] showed significantly better results after 3 months compared to baseline. However, follow-up was not performed for longer than 3 months, so it is not possible to translate these results to the long-term.

Treatment with MT on the TMJ seemed to lose its impact on MMO after 6 months [21]. By itself, a regression towards baseline was observed, which continued until 12-month follow-up, suggesting the importance of the active part of treatment for range of motion (ROM) increase.

It is worth pointing out that MT on the cervical region is suggested to have no effect on MMO [14]. No change in any of the groups was reported in the follow-up periods of any study. This is in contrast to the pain outcome, which showed an improvement during the first few weeks. These results may be explained by the complexity of this pathology and of the pain mechanism themselves, as a limitation in mouth opening could be related to purely biomechanical problems; therefore, in the absence of treatment focused specifically on the area, no significant improvement is achieved.

### 4.3. Study Characteristics

The patients from the included studies were relatively homogeneous. The included patients reflected the usual characteristics of TMD, with a higher incidence in middle-aged adults and being 1.5 to 2.5 times more frequent in women than men [4].

The treatment sessions were performed with varying frequencies and durations. Both were dependent on the type of treatment used, as manipulations were performed in a shorter time with a greater interval between sessions (one session every 2 weeks, no session time established), while joint mobilizations were usually carried out more frequently and for a longer time during each session (two sessions per week, from 15 to 45 min each). It should be noted that in addition to manipulative therapy, subjects also performed daily exercises, some of them as frequently as once per hour. Although MT was used less frequently, all the patients carried out a TE program every day.

The MT treatments themselves also varied. Regarding the muscular system, the masseter and temporal muscles were most often targeted for treatment, while only one study [21] also treated the lateral and medial pterygoids. It is interesting to note that only two of the studies [20,21] adoptedan intraoral approach during treatment. Future studies should analyze whether the obtained results may be partly due to this kind of treatment.

### 4.4. Strengths and Weaknesses

In our opinion, the main strength of this review is that, to the best of our knowledge, it is the first to collate and summarize the medium- and long-term results of using MT for TMD, and also consider whether TE was involved or not.

The weaknesses of the review are related to the number of databases used. Although the search was carried out using five databases with high dissemination (PubMed, SCOPUS, Cochrane, SciELO and PEDro), we cannot exclude the possibility that other articles exist that met the established criteria but were indexed in a smaller database. At the same time, only English, Spanish, Portuguese and French documents were included, excluding any other possibly adaptable articles that met the remaining criteria.

The differences in the results obtained for MT and TE compared to other therapies, such as splints or education, are not well defined. The low number of long-term follow-up studies, together with the wide variability in the techniques used and moderate methodological quality, suggest that further research with more treatments and higher quality are needed for carrying out a more comprehensive analysis. Our results are in line with those observed for other musculoskeletal pathologies [15,25,26], in which MT, TE and patient education all seem to improve pain, disability and quality of life in the medium and long term.

Finally, the number of studies that carried out medium- and long-term follow-ups were insufficient to be able to carry out a meta-analysis, so the changes experienced immediately after the intervention and in the short term were presented (Figure 2 and Figure 3, respectively).

## 5. Conclusions

After analyzing the available literature, we can conclude that MT seems to be an effective treatment for TMD in the medium term, although the effect appears to decrease over time. However, when complemented with TE, these effects can be maintained in the long term. It is important to emphasize the limited number of articles that met the set criteria for the current review.

## Figures and Tables

**Table 1:** Methodological quality according to PEDro scale.

	Garrigós-Pedrón M. (2018) [14]	Nagata K. et al. (2018) [19]	Craane B. et al. (2012) [20]	Guarda-Nardini L. et al. (2011) [18]	Kalamir A. et al. (2011) [21]	Ismail F. et al. (2007) [22]
Item 1	●	●	●	●	●	●
Item 2	-	●	●	-	●	-
Item 3	●	●	●	●	●	●
Item 4	-	●	-	-	-	-
Item 5	-	-	-	-	-	-
Item 6	●	-	●	-	●	-
Item 7	●	●	●	-	●	●
Item 8	-	●	●	-	●	-
Item 9	●	●	●	●	●	●
Item 10	●	●	●	●	●	●
TOTAL	6/10	8/10	8/10	4/10	8/10	5/10

Items: 1 = Subjects were randomly allocated to groups; 2 = Concealed allocation; 3 = Groups were similar at baseline; 4 = Blinding of all subjects; 5 = Blinding of all therapists; 6 = Blinding of all assessors; 7 = Measures for at least one key outcome were obtained from more than 85% of the subjects; 8 = Intention to treat analysis; 9 = Results of between-group statistical comparisons reported for at least one key outcome; 10 = Provides both point measures and measures of variability for at least one key outcome.● = present item

**Table 2:** Included studies’ features.

Author	Sample Size (Women)	Age	Pathology	Interventions	Session Frequency
Garrigós-Pedrón M. et al. (2018)	45(39) CG:22(19) COG:23(20)	CG: 48.2 (±11.3) COG: 46.0 (±9.1)	Chronic migraine, myofascial TMD	CG = TE (cervical muscle isometric exercises, neurodynamics) + MT (suboccipital muscle inhibition technique, passive mobilization) COG = CG + caudal longitudinal technique, NMT, TMJ TE, neurodynamics	6 sessions 30 min/session 3–6 weeks
Nagata K. et al. (2018)	61(50) TE+MT: 31 TE: 30	49.6 (±25) TE+MT: 48.2 (±21.1) TE: 50.7 (±18.3)	TMD, myalgia and/or arthralgia during mouth opening or palpation, limited opening (<35mm): “non-reducing disc displacement with opening limitation”	TE+MT = “boost”-type manipulation + ET TE = self-exercises, CBT, education	MT: 1 session/2 weeks (until >40mm MMO) TE: opening exercises 10 rep. 3–5/day, SMFT every hour
Craane B. et al. (2012)	49(49) Physio: 23(23) Control: 26(24)	Physio: 34.7 (±14) Control: 38.5 (±15.1)	Non-reducing disc displacement with opening limitation	Physio= Education, temporal muscle stretching, intraoral treatment of masseter, opening exercises, traction and opening mobilization, self-treatment Control = Education	Physio: 2 sessions/week during first 3 weeks; 1 session/week during last 3 weeks
Guarda-Nardini L. et al. (2011)	30(22) BT:15(11) FMT: 15(11)	BT: 47.7(±14.3) FMT: 43.2(±13.9)	Myofascial TMD with or without pain during mouth opening, for at least 6 months	BT = Several BT injections in masseter and temporal muscles FMT = Manual pressure at craniocervical coordination centers	BT: 1 session FMT: 3 (±1) sessions 50 min 2–4 weeks
Kalamir A. et al. (2011)	93(50) IMT: 31(17) IMTES: 31(16) Control: 31(17)	IMT: 35(±6.7) IMTES: 34(±6.1) Control: 35(±5.0)	Daily periarticular pain with or without noises, for at least 3 months	IMT = Intraoral pressure in temporal muscle, lateral and medial pterygoid muscles, intraoral sphenopalatine ganglion IMT+E+S = IMT + education + self-care (cross-pressure chewing technique, post-isometric) Control = No treatment (waiting list)	IMT: 2 sessions/week 10–15 min/session 5 weeks IMTES: 4 sessions Educative talk for 2 min Exercises 2 times/day
Ismail F. et al. (2007)	26(23) G1: 13(10) G2: 13(13)	G1: 44.5(±14.1) G2: 41.7(±16.5)	Arthrogenicacute TMD with less than 6 months evolution, <38 mm MMO with pain	Group 1 = Michigan splint 24 h/day except during meals Group 2 = Michigan splint + TMJ traction mobilization, massage therapy	2 sessions/week 45 min/session 12 weeks

BT: botulinum toxin; CG: control group; COG: cervical and orofacial group; CBT: cognitive-behavioral therapy; FMT: fascial manipulation technique; IMTES: intraoral myofascial therapies + educational selfcare exercises; IMT: intraoral myofascial therapies; MMO: Maximal Mouth Opening; MT: manual therapy; NMT: neuro-muscular techniques; SMFT: simplified myo-functional therapy; TE: therapeutic exercise; TMJ: temporomandibular joint; TMD: temporomandibular disorders.

**Table 3:** Represented results for outcome evolution are those from groups treated with MT. In the case of pain not being measured with VAS, it will be referenced by using a percentage (0–100).

Author	Outcomes (Main; *Secondary*)	Follow-Up	Outcome Evolution									
			Pain (VAS)					MMO (mm)				
			Base	Post	3 M	6 M	1 Y	Base	Post	3 M	6 M	1 Y
Garrigós-Pedrón M. (2018)	CF-PDI, HIT-6; TSK-11, VAS, temporal; masseter muscle and wrist PPT; pain-free active MMO	6 weeks 3 months	7.63 (1.16)	5.965 (1.426) ** d = 1.31	4.126 (2.150) ** d = 2.75	−	−	32.87 (7.16)	37.22 (5.98) ** d = −0.61	41.13 (6.49) ** d = −1.15	−	−
Nagata K. (2018)	Active MMO, pain and noises from TMJ using NRS 0–10	Every 2 weeks, for 18 weeks	5.76 (2.26)	GRAF	GRAF	−	−	28.32 (4.55)	GRAF	GRAF	−	−
Craane B. (2012)	MPQ, VAS, MFIQ, active and passive MMO (mm), masseter and temporal muscle PPT	3, 6, 12, 26, 52 weeks	5.0 (3.8–6.0)	−	2.3 (0.65–3.35)	1.75 (0.05–3)	0.2 (0–1.6)	35.8 (7.4)	−	39.4 (6.3)	41.6 (6)	42.7 (5.7)
Guarda-Nardini L. (2011)	VAS, ROM	3 months	6.0 (2.0)	2.1 (1.4) *	2.5 (2.2) *	−	−	52.0 (9.5)	−	52.4 (N.S.)	−	−
Kalamir A. (2011)	0–11 scale for pain at rest, MMO, plus both of them while clenching their jaws; *MMO (mm), GRC 0–7*	6 weeks 6 months 1 year	4.46 (6.41) = 4.05(5.82) 4.26 (7.49) = 3.87(6.81)	−	− (N.S.)	GRAF	−3.1 = −2.82 −4.0 = −3.6	37.42 39.89	−	−	GRAF	GRAF
Ismail F. (2007)	Active and passive MMO (mm), protrusion movement (mm), VAS (total, during movement, at rest, after mandibular loading)	1 week 1 month 6 weeks 3 months	4.5 (2)	−	−2.8 (2.1)	−	−	30.1 (5.4)	−	40.8 (4.1)	−	−

CF-PDI: Cranio-Facial Pain and Disability Inventory; HIT-6: Headache Impact Test; TSK-11: Tampa Scale for Kinesiophobia; PPT: Pain Pressure Threshold; MMO: Maximal Mouth Opening; NRS: Numeric Rating Scale; MPQ: McGuil Pain Questionnaire; MFIQ: Migraine Functional Impact Questionnaire; GRC: Global Reporting of Change; N.S.: Non-significant results; *: *p*< 0.05; **: *p*< 0.001; GRAF: Results from the original document were shown in graphical form only, resulting in an inability to quantify them accurately.; ROM: range of motion; VAS: visual analogue scale.

## References

[1] Isberg A. (2001). Temporomandibular Joint Dysfunction: A Practitioner’s Guide.

[2] Akheel D.M. (2014). Temporomandibular Joint Disorders.

[3] Perez C.V., de Leeuw R., Okeson J.P., Carlson C.R., Li H.-F., Bush H.M., Falace D.A. (2013). The incidence and prevalence of temporomandibular disorders and posterior open bite in patients receiving mandibular advancement device therapy for obstructive sleep apnea. Sleep Breath. Schlaf. Atm..

[4] Machado L.P.e.S., de Nery C.G., Leles C.R., de Nery M.B.M., Okeson J.P. (2009). The prevalence of clinical diagnostic groups in patients with temporomandibular disorders. Cranio J. Craniomandib. Pract..

[5] Peck C.C., Goulet J.-P., Lobbezoo F., Schiffman E.L., Alstergren P., Anderson G.C., de Leeuw R., Jensen R., Michelotti A., Ohrbach R. (2014). Expanding the taxonomy of the diagnostic criteria for temporomandibular disorders. J. Oral Rehabil..

[6] Isberg A., Stenstrom B., Isacsson G. (1991). Frequency of bilateral temporomandibular joint disc displacement in patients with unilateral symptoms: A 5-year follow-up of the asymptomatic joint. A clinical and arthrotomographic study. Dento Maxillo Facial Radiol..

[7] Okeson J.P., de Leeuw R. (2011). Differential diagnosis of temporomandibular disorders and other orofacial pain disorders. Dent. Clin. North Am..

[8] Pesquera Velasco J., Casares García G., Jiménez Pasamontes N., García Gómez F.A. (2005). Método de ayuda para el diagnóstico de los trastornos de la articulación temporomandibular: Análisis discriminante aplicado a los Trastornos Temporomandibulares. Med. Oral Patol. Oral Cir. Bucal Ed. Impresa.

[9] Tjakkes G.-H.E., Reinders J.-J., Tenvergert E.M., Stegenga B. (2010). TMD pain: The effect on health related quality of life and the influence of pain duration. Health Qual. Life Outcomes.

[10] Roldán-Barraza C., Janko S., Villanueva J., Araya I., Lauer H.-C. (2014). A systematic review and meta-analysis of usual treatment versus psychosocial interventions in the treatment of myofascial temporomandibular disorder pain. J. Oral Facial Pain Headache.

[11] Fricton J. (2006). Current evidence providing clarity in management of temporomandibular disorders: Summary of a systematic review of randomized clinical trials for intra-oral appliances and occlusal therapies. J. Evid.-Based Dent. Pract..

[12] Butts R., Dunning J., Pavkovich R., Mettille J., Mourad F. (2017). Conservative management of temporomandibular dysfunction: A literature review with implications for clinical practice guidelines (Narrative review part 2). J. Bodyw. Mov. Ther..

[13] De Gomes C.A.F.P., Politti F., Andrade D.V., de Sousa D.F.M., Herpich C.M., Dibai-Filho A.V., de Gonzalez T.O., Biasotto-Gonzalez D.A. (2014). Effects of massage therapy and occlusal splint therapy on mandibular range of motion in individuals with temporomandibular disorder: A randomized clinical trial. J. Manip. Physiol. Ther..

[14] Garrigós-Pedrón M., La Touche R., Navarro-Desentre P., Gracia-Naya M., Segura-Ortí E. (2018). Effects of a Physical Therapy Protocol in Patients with Chronic Migraine and Temporomandibular Disorders: A Randomized, Single-Blinded, Clinical Trial. J. Oral Facial Pain Headache.

[15] Wewege M.A., Booth J., Parmenter B.J. (2018). Aerobic vs. resistance exercise for chronic non-specific low back pain: A systematic review and meta-analysis. J. Back Musculoskelet. Rehabil..

[16] Martins W.R., Blasczyk J.C., de Aparecida Furlan Oliveira M., Lagôa Gonçalves K.F., Bonini-Rocha A.C., Dugailly P.-M., de Oliveira R.J. (2016). Efficacy of musculoskeletal manual approach in the treatment of temporomandibular joint disorder: A systematic review with meta-analysis. Man. Ther..

[17] Armijo-Olivo S., Pitance L., Singh V., Neto F., Thie N., Michelotti A. (2016). Effectiveness of Manual Therapy and Therapeutic Exercise for Temporomandibular Disorders: Systematic Review and Meta-Analysis. Phys. Ther..

[18] Guarda-Nardini L., Stecco A., Stecco C., Masiero S., Manfredini D. (2012). Myofascial pain of the jaw muscles: Comparison of short-term effectiveness of botulinum toxin injections and fascial manipulation technique. Cranio J. Craniomandib. Pract..

[19] Nagata K., Hori S., Mizuhashi R., Yokoe T., Atsumi Y., Nagai W., Goto M. (2019). Efficacy of mandibular manipulation technique for temporomandibular disorders patients with mouth opening limitation: A randomized controlled trial for comparison with improved multimodal therapy. J. Prosthodont. Res..

[20] Craane B., Dijkstra P.U., Stappaerts K., De Laat A. (2012). Randomized controlled trial on physical therapy for TMJ closed lock. J. Dent. Res..

[21] Kalamir A., Bonello R., Graham P., Vitiello A.L., Pollard H. (2012). Intraoral myofascial therapy for chronic myogenous temporomandibular disorder: A randomized controlled trial. J. Manip. Physiol. Ther..

[22] Ismail F., Demling A., Hessling K., Fink M., Stiesch-Scholz M. (2007). Short-term efficacy of physical therapy compared to splint therapy in treatment of arthrogenous TMD. J. Oral Rehabil..

[23] Huang H., Grant J.A., Miller B.S., Mirza F.M., Gagnier J.J. (2015). A Systematic Review of the Psychometric Properties of Patient-Reported Outcome Instruments for Use in Patients with Rotator Cuff Disease. Am. J. Sports Med..

[24] Delitto A., George S.Z., Van Dillen L., Whitman J.M., Sowa G.A., Shekelle P., Denninger T.R., Godges J.J. (2012). Low back pain: Clinical linked to the international classification of functioning, disability and health from the orthopaedic section of the American Physical Therapy Association. J. Orthop. Sports Phys. Ther..

[25] Martinez-Calderon J., Flores-Cortes M., Morales-Asencio J.M., Luque-Suarez A. (2020). Conservative Interventions Reduce Fear in Individuals with Chronic Low Back Pain: A Systematic Review. Arch. Phys. Med. Rehabil..

[26] Ulger O., Demirel A., Oz M., Tamer S. (2017). The effect of manual therapy and exercise in patients with chronic low back pain: Double blind randomized controlled trial. J. Back Musculoskelet. Rehabil..

